# Usefulness of molecular biology performed with formaldehyde-fixed paraffin embedded tissue for the diagnosis of combined pulmonary invasive mucormycosis and aspergillosis in an immunocompromised patient

**DOI:** 10.1186/1746-1596-5-1

**Published:** 2010-01-08

**Authors:** Véronique Hofman, Abdelmajid Dhouibi, Catherine Butori, Bernard Padovani, Martine Gari-Toussaint, Dea Garcia-Hermoso, Michèle Baumann, Nicolas Vénissac, Gieri Cathomas, Paul Hofman

**Affiliations:** 1 Laboratory of Clinical and Experimental Pathology, Louis Pasteur Hospital, 30 avenue de la voie romaine, Nice, 06002, France; 2 Human Biobank, Louis Pasteur Hospital, 30 avenue de la voie romaine, Nice, 06002, France; 3 Department of Radiology, Louis Pasteur Hospital, 30 avenue de la voie romaine, Nice, 06002, France; 4 Laboratory of Mycology, Archet II Hospital, 151 route Saint-Antoine de Ginestière, Nice, 06200, France; 5 Centre National de Référence Mycologie et Antifongiques (CNRMA); CNRS URA3012, Molecular Mycology Unit, Pasteur Institute, 25 rue du Docteur Roux, Paris, 75015, France; 6 Laboratory for Molecular and Infectious Disease Pathology, Cantonal Institute for Pathology, Mühlemattstrasse 11, Liestal, 4410, Switzerland; 7 Department of Thoracic Surgery, Louis Pasteur Hospital, 30 avenue de la voie romaine, Nice, 06002, France

## Abstract

Immunocompromised patients who develop invasive filamentous mycotic infections can be efficiently treated if rapid identification of the causative fungus is obtained. We report a case of fatal necrotic pneumonia caused by combined pulmonary invasive mucormycosis and aspergillosis in a 66 year-old renal transplant recipient. *Aspergillus *was first identified during the course of the disease by cytological examination and culture (*A. fumigatus*) of bronchoalveolar fluid. Hyphae of *Mucorales *(*Rhizopus microsporus*) were subsequently identified by culture of a tissue specimen taken from the left inferior pulmonary lobe, which was surgically resected two days before the patient died. Histological analysis of the lung parenchyma showed the association of two different filamentous mycoses for which the morphological features were evocative of aspergillosis and mucormycosis. However, the definitive identification of the associative infection was made by polymerase chain reaction (PCR) performed on deparaffinized tissue sections using specific primers for aspergillosis and mucormycosis. This case demonstrates that discrepancies between histological, cytological and mycological analyses can occur in cases of combined mycotic infection. In this regard, it shows that PCR on selected paraffin blocks is a very powerful method for making or confirming the association of different filamentous mycoses and that this method should be made available to pathology laboratories.

## Introduction

Invasive fungal infections appear to have increased over the past few years, mostly in immunocompromised patient [1-4]. Moreover, the diagnosis of invasive filamentous fungal disease (IFFD) is often difficult in immunodeficient patients. Tissue specimens are difficult to obtain in debilitated patients, but are essential in detecting invasive fungi [5]. In the absence of identification of fungal elements in tissue, the diagnosis of a "probable" invasive fungal disease (IFD) can be retained when a host factor, clinical features and mycological evidence are present [5]. Additionally, the diagnosis of a "possible" IFD is made when appropriate host factors and sufficient clinical evidence are present [5]. Since a high level of mortality occurs in immunocomprised patients with IFFD [6-8], an accurate and rapid diagnosis is required in order to give the appropriate anti-fungal treatment. In addition, depending of the isolated fungal species, some of these treatments can be inefficient [9,10]. The combination of two or more IFFD occurring simultaneously or sequentially in immunocompromised patients makes diagnosis of these patients very challenging. Combined invasive mucormycosis and aspergillosis has been rarely described, its occurrence has probably been underestimated [11-16]. Predisposing factors and clinical features in association with these mycoses are almost the same [17,18]. Although mycological analyzes allow definitive identification of the different species, combined infections are diagnosed histologically.

We report a case of an associated aspergillosis and mucormycosis infection responsible for fatal necrotic and extensive pneumonia in a patient with a renal transplant. Mycological analysis identified *Aspergillus fumigatus *in a culture of bronchoalveolar lavage (BAL) and *Rhizopus microsporus *in a culture of a lung tissue specimen. Identification of a combined aspergillosis and mucormycois infection was made by PCR performed on formaldehyde pulmonary fixed tissues.

## Case report

**Figure 1:**
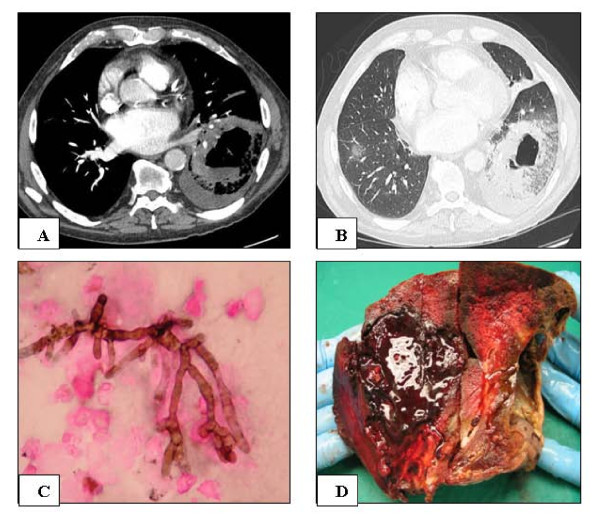
**A and B: CT scan showing a large excavated pulmonary mass of the pulmonary left inferior lobe, outlined by a glassy feature and associated with pleural effusion (A. Non injected CT scan; B Injected CT scan)**. **C**. Mycotic filaments showing the morphological features of aspergillosis isolated from the BAL (Gomori-Grocott silver method × 1000). **D**. Gross macroscopy of the resected pulmonary lobe showing a hemorrhagic infarct.

A 66-year-old man, who had received a renal transplant 5 months before, and was on tritherapy [prednisolone (1 mg/kg/day); cyclosporine A (7 mg/kg/day); azathioprine (1 mg/kg/day)], was admitted to the hospital with hemoptysia. A computed tomographic scan of the chest showed a not well-circumscribed and excavated mass mass involving the pulmonary inferior left lobe, bilateral pleural effusions, and a small parenchyma nodule in the pulmonary inferior right lobe (Fig. 1A et 1B). An analysis of the BAL was quickly performed due to the presence of *Aspergillus fumigatus *in sputum: cytopathological examination showed filamentous hyphae corresponding to aspergillosis (Fig. 1C). These filaments were subsequently identified by culture as *A. fumigatus*. In addition, pyocyanic bacteria were isolated from the BAL. Treatment associating voriconazole, ceftazidime and ciprofloxacine was started. However, the absence of a clinically improved status and repeated massive hemoptysia, led to surgical resection of the pulmonary left inferior lobe one week later. Gross macroscopy demonstrated a diffuse hemorrhagic infarct of the lung (Fig. 1D). A specimen of lung tissue was sent to the mycology laboratory. The patients died of cardio-pulmonary failure two days after surgery.

## Materials and methods

### Histological study

Numerous specimens (more than thirty) were taken from the surgical specimen, fixed in 10% formaldehyde and paraffin embedded. 5 μm paraffin sections were stained with haematoxylin eosin safran (HES), periodic acid Schiff (PAS), Gomori Grocott's methanamine silver (GGM), Gram, Warthin-Starry, and Zielh-Neelsen stains.

### Molecular biology

Tissue sections were deparaffinized with xylene and DNA was extracted from formalin fixed, paraffin embedded tissue using QIAmp DNA Mini Kit (QIAGEN AG, Hombrechtikon, Switzerland) as described by the manufacturer. The presence of adequate DNA was confirmed by amplifying the human beta-globin gene as previously described [19]. Previously described specific sequences of 5.8 sRNA and 18 sRNA of *Aspergillus sp*. and mucor, respectively, were chosen as target sequences with slight modification [20,21]. For the detection of mucor, nested PCR was used with primers described previously [20], but with modification: Mucor1 (5'-WTTACC RTG AGC AAA TCA GA-3') and Mucor2 (5'-CAA TCY AAG AAT TTC ACC TCTAG-3) for the first round and Mucor3 (5'-AGC ATG GAA TAA TRA AAY A-3') and Mucor4 (5'-AGC ATG GGA TAA CGG AAT A-3) for the second round, respectively, giving a final PCR product of 124 bp. For the detection of Aspergillus, the following primers were used as described [21], but with modification: AspNest1 (5'-TCTTGGTTCCGGCATCGAT-3) and AspNest2 (5'TGACAAAGCCCCATACGCT-3') for the first round and AspNest3 (5'-GAAGAACGCAGCGAAATGC-3') and AspNest4 (5'-AACACACAAGCCGTGCTTGA-3') for the second round, respectively, leading to a final PCR product of 146 bp. The basic amplification reactions were done as described before using a volume of 50 μl containing 100 to 500 ng of DNA, 2.5 ml of each primer (final concentration 3 mM), 25 μl Master Mix Gold (Applied Biosystems, Paris, France) [22]. Amplification and detection were performed on the ABI 9800 Fast Thermal Cycler (Applied Biosystem) using for both primer sets, the same cycling conditions with an initial cycling step at 94°C for 10 minutes followed by 94°C, 55°C and 72°C for 30 seconds each ending at 4°C. Ten microliters of each amplification product was analyzed by electrophoresis in an ethidium bromide-containing 2% agarose gel. In addition, PCR products were extracted from a low melting agarose gel and DNA sequencing was performed to confirm the appropriate DNA sequence. Stringent laboratory conditions and appropriate negative and positive controls were performed in each run.

### Mycological analysis

The microscopic examination of the BAL was done in a mixture of potassium hydroxide and chlorazole black E and demonstrated septate and dichotomous branching hyphae. *A. fumigatus *grew on Sabouraud's glucose agar at 28°C. The strain was tested against voriconazole with the E-test method and showed a good sensitivity [Minimum Inhibit Concentration = 0.125 μg/ml]. The direct examination of the per operative pulmonary biopsy showed non-septate right-angled -branching hyphae suggestive of *Mucorales*.

## Results

### Pathological findings

**Figure 2:**
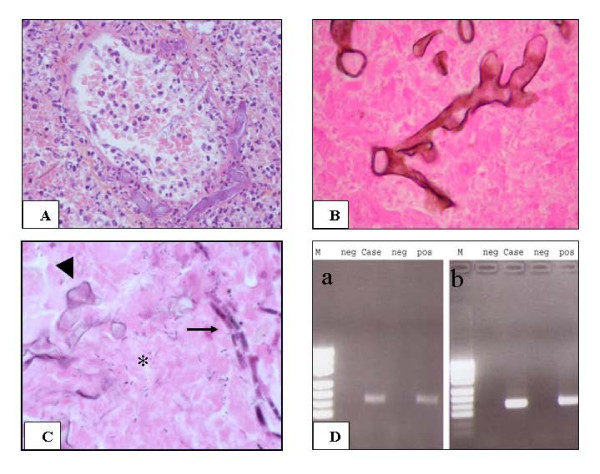
**A. Hyphae with a large diameter invading blood vessels (HES, original magnification, × 400)**. **B**. Morphological features of mucorales (Gomori-Grocott silver method, original magnification × 1000). **C**. Association of two different hyphae in the same tissue section showing morphological feature of mucorales (arrowhead) and of aspergillosis (arrow). Asteriks: associated bacteria (HES, original magnification, × 800). **D**. Molecular biology **a**: PCR of Aspergillus **b**: PCR of Mucor: M: Marker, neg: negative controls, pos: positive controls.

All tissue sections stained with HES showed large areas of infarct associated with a few neutrophils, histiocytes and lymphocytes. Venules, arteries and capillaries were congestive or thrombotic. Most of these blood vessels were invaded by large hyphae, measuring from 30 to 50 μm in their largest diameter (Fig. 2A). GGM staining confirmed the presence of numerous fungal elements indicative of a mucormycosis infection: presence of broad-based non septated hyphae with branching at right angles, or twisted hyphae (Fig. 2B). The hyphae were faintly stained with PAS. In a few areas, two different subtypes of hyphae were found in association: first some hyphae were identical to those described above, and second some thinner septated hyphae, 8 to 12 μm in diameter, regular, sometimes branching at acute angles, which could correspond to aspergillosis (Fig. 2C). These filaments invaded blood vessels. Numerous bacteria were observed on HES and Gram staining (Fig. 2C). Other stains gave negative results.

PCR analysis from deparaffinized sections performed from a selected paraffin block showing both subtype of hyphae, allowed identification of aspergillosis and mucormycosis (Fig. 2D). DNA sequencing of the PCR products were performed and showed a 100% concordance with *Aspergillus sp*. and *Rhizopus sp*. (namely *Rhizopus microsporus*) for the apergillus and mucor PCR, respectively.

### Mycological findings

**Figure 3:**
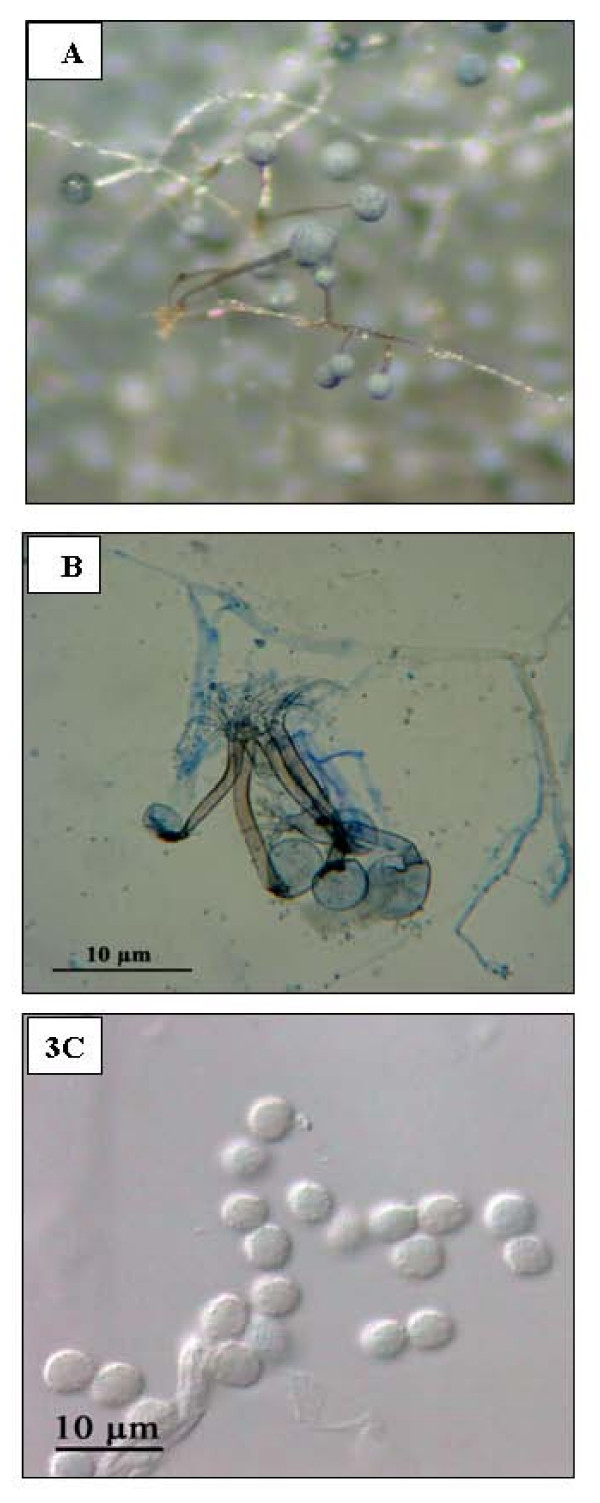
**A. Stereomicroscopic image of the habit sketch of *Rhizopus microsporus *var *rhizopodiformis***. **B**. Micromorphology showing the dark brown unbranched sporangiophores born directly from rhizoids. **C**. Finely roughened (sub) spherical sporangiospores

Culture on Sabouraud's dextrose agar at 28°C yielded a fast-growing mold (3 days). Colonies were woolly and initially white, quickly become grayish brown. The fungus was identified as *Rhizopus microsporus *var. *rhizopodiformis *on the basis of morphological features: rhizoids and sporangiophores of 400 μm in length and in groups of 1 to 4; sporangia spherical of approximately 100 μm grayish black; spherical sporangiospores (5 μm) fine spinulose and the capacity to grow at 50°C (Fig. 3A-C). The morphological identification was confirmed by sequencing the whole ITS1-5.8S-ITS2 region [23]. Antifungal sensitivity testing using the EUCAST method showed a decreased susceptibility to the voriconazole (MIC = 8 μg/ml); and a MIC that was considered to be within the sensitivity range to amphotericin B (MIC = 0.06 μg/ml) and posaconazole (MIC = 0.5 μg/ml).

## Discussion

Although invasive mycoses have long been recognized for a long time as being caused by significant pathogens, particularly in immunocompromised patients, the frequency of infection with opportunistic fungi is increasing with time and the spectrum of the infectious agent of invasive mycoses is changing [1-4]. In this regard, advances in therapeutic technologies and in particular the development of novel immunosuppressive therapies, have prolonged the period of risk for many individuals [3,4]. A large number of invasive mycoses have been identified because of the increase in the number of patients at risk [24-26]. Most of the invasive mycoses are caused by filamentous fungi, rarely by other fungi [6,27-29]. In this regard, a number of IFFD has been reported in renal transplant patients, particularly aspergillosis and mucormycosis [30]. Other IFFD have been more rarely described in immunocompromised hosts, such as scedosporiosis [31].

A confirmed diagnosis of IFFD is made on the demonstration of hyphae in the diseased tissue. Pathological identification of the hyphae is based on different well-described criteria: diameter, presence of septa, and branching angle (right or acute branching), pigmentation [32,33]. It is important to consider the characteristics of the infiltrate at the inflammatory region that is in contact with hyphae: presence of angioinvasion, of giant cells and of eosinophilic necrosis [34]. Mycological analysis made on non fixed-tissue specimens, associating microscopic examination and culture allows identification of the species and the study of the sensitivity to anti-fungal drugs. However, the different results of these mycological analyses can be negative in the following circumstances: 1) When there is an error in sampling, the specimen sent to the mycology laboratory does not contain the different fungi, 2) When the specimen containing the hyphae is totally necrotic. In addition, when two filamentous mycoses are present in the same organ, the mycological results are even more inconstant, since only one fungus might be present on the specimen sent to the mycology laboratory. In these different situations, it is essential to compare the culture results and the morphological analysis of the tissue sections. However, histological analyses have some limits, even after histochemical staining.

The distinction between aspergillosis and scedosporiosis, between aspergillosis and fusariosis and certain mucormycosis, such as those caused by *Cunninghamella *from tissue sections may be difficult or impossible [31,35,36]. Other potential pitfalls are more easily eliminated [37,38]. Ancillary methods using specific antibodies raised against filamentous fungi can be helpful for diagnosis. However, the different antibodies are rarely commercially available [33,36,39]. Finally, *in situ *hybridization has been developed for fungal detection in fixed-tissue sections, but the probes used for this method are rarely available in the pathology laboratory [40]

In recent years, PCR methods performed with deparaffinized tissue sections have been used to try to improve the detection and identification of pathogens in fixed specimens [39,41]. This methodology needs to use strict positive and negative controls and exact comparison with histological features and mycological analysis is required. However, false-negative and false-positive results due to artefacts in amplification, or sampling, and possible exogenous contamination by microorganisms, still are very important pitfalls in molecular diagnostic pathology [42,43]. In the present case, there was no evidence of contamination by external sources and strict laboratory precautions were applied to avoid carry over and false positive results.

The present case report underlines the usefulness of the PCR method performed with embedded paraffin fixed specimens, which allows simultaneous detection on the same tissue of two types of IFFD. Moreover, the pathogenic status of these pathogens can be confirmed by comparing histopathological and molecular biology results obtained from the same embedded paraffin tissue. In the present case, the absence of mycological diagnosis of aspergillosis from non-fixed biopsy specimen was probably due to an error in sampling. In this regard, histological analysis showed only a few areas with the two associated mycoses. Filaments of aspergillosis, but not of mucorales, were noted by cytopathological analysis of the BAL and identified as *A. fumigatus *in culture. This can be due to an error in sampling, but also because at that time mucorales was undetectable, since it grew subsequently in the patient treated with voriconazole. However, the clinical distinction between aspergillosis and mucormycosis is crucial since there is an increased incidence of mucormycosis in patients treated with voriconazole for suspected aspergillosis [44]. In this regard, a couple of cases of mucormycosis occurring in patients with voriconazole-treated aspergillosis have been reported [9,44]. The diagnosis of IFFD based on hyphae isolated from the BAL is uncertain. In this case, different associated clinical features and predisposing host factors must be present before giving specific treatment, which can be toxic, ineffective, and favor the development of another mycotic disease [45,46]. Finally, for most clinicians, the identification of aspergillosis or mucormycosis in the BAL is not sufficient to confirm the diagnosis of IFFD [47]. The utility of performing transbronchial or transparietal biopsies in debilitated patients should be discussed [48,49].

## Conclusion

Our observations outline the difficulty of diagnosing associated invasive mycoses in immunocompromised patients. For diagnosis, it is crucial to compare the histological and mycological analyses. However, as demonstrated here, molecular biology performed on FFPE tissue sections, after morphological control on a mirrored stained tissue section, can be the only method for the identification of different pathogens in case of discrepancies between pathological and mycological approaches. In this regard, we strongly believe that molecular biology of FFPE tissues should be developed and available to surgical pathology laboratories for infectious diseases pathology, since, in particular in immunocompromised patients, it is crucial in cases of invasive mycotic diseases to provide a rapid and optimal treatment

## Competing interests

The authors declare that they have no competing interests.

## Authors' contributions

VH was involved in the histopathological evaluation and interpreted the histopathology. AD and CB performed the literature search, prepared the materials and supplied the relevant clinical details. BP provided the radiological details. MGT and DGH interpreted the mycology data. MB was involved in molecular biology experiments. GC interpreted the molecular biology data from paraffin sections. NV was involved in surgery. PH outlined the general concept and was involved in drafting and revising the manuscript. All authors have read and approved of the present manuscript.
